# The Rationale for Sulforaphane Favourably Influencing Gut Homeostasis and Gut–Organ Dysfunction: A Clinician’s Hypothesis

**DOI:** 10.3390/ijms241713448

**Published:** 2023-08-30

**Authors:** Christine A. Houghton

**Affiliations:** 1 Institute for Nutrigenomic Medicine, Cleveland, QLD 4163, Australia; christine.houghton@nutrigenomicmedicine.com; Tel.: +617-3488-0385; 2 Cell-Logic, 132-140 Ross Court, Cleveland, QLD 4163, Australia

**Keywords:** sulforaphane, gut–organ axis, gut ecology, microbiome, chronic disease, nutrigenomics, nutritional medicine, food intolerance, epithelium, dysbiosis, gut barrier

## Abstract

Given the increasing scientific, clinical and consumer interest in highly prevalent functional gastrointestinal disorders, appropriate therapeutic strategies are needed to address the many aspects of digestive dysfunction. Accumulating evidence for the crucifer-derived bioactive molecule sulforaphane in upstream cellular defence mechanisms highlights its potential as a therapeutic candidate in targeting functional gastrointestinal conditions, as well as systemic disorders. This article catalogues the evolution of and rationale for a hypothesis that multifunctional sulforaphane can be utilised as the initial step in restoring the ecology of the gut ecosystem; it can do this primarily by targeting the functions of intestinal epithelial cells. A growing body of work has identified the colonocyte as the driver of dysbiosis, such that targeting gut epithelial function could provide an alternative to targeting the microbes themselves for the remediation of microbial dysbiosis. The hypothesis discussed herein has evolved over several years and is supported by case studies showing the application of sulforaphane in gastrointestinal disorders, related food intolerance, and several systemic conditions. To the best of our knowledge, this is the first time the effects of sulforaphane have been reported in a clinical environment, with several of its key properties within the gut ecosystem appearing to be related to its nutrigenomic effects on gene expression.

## 1. Introduction

### Author’s Preamble

As an experienced clinician in Nutritional Medicine [1], and more recently a researcher on the nutrigenomic effects of phytochemicals, I have observed remarkable clinical responses to the crucifer-derived molecule sulforaphane (SFN). So significant are these responses that I have catalogued my findings, and 4 years ago, I conveyed these and my methods of implementation to other clinicians within my sphere of influence. This approach soon revealed that SFN exhibits beneficial effects on the gut as a functional ecosystem made up of the gut epithelium, its underlying immune network, and the microbial inhabitants of the gut lumen. In outlining the role of SFN in this hypothesis, it is essential that we provide the framework for the hypothesis by first discussing key elements of these three components of the gut ecosystem.

Functional gastrointestinal disorders (FGIDS) are among the most common and unresponsive conditions that present to clinicians, in both the medicine and dietetics–nutrition disciplines. A 2021 large-scale multinational study found that more than 40% of persons worldwide experience FGIDs [2].

Irritable Bowel Syndrome (IBS), as a sub-category of FGIDS, possesses a global prevalence of 15–20% [3], and can be grouped into three sub-categories: IBS-C (constipation), IBD-D (diarrhoea), IBS-M (mixed). The recommended treatments are very broad, and cover psychotherapeutic, dietary and pharmaceutical methods; the use of probiotics and other supplements is more recent. However, the benefits are inconsistent, and the use of medication strongly debated [4]. Clearly, a better understanding of the mechanisms that underpin IBS is necessary, and as a clinician who has worked with such challenging patients, I am motivated by a strong desire to improve clinical outcomes.

The demand for solutions to digestive health issues is increasing, especially since both the scientific literature and the popular press have started dedicating significant resources to promoting awareness of what has come to be known as ‘gut health’. In a quest to find solutions to their IBS and other digestive health issues, consumers have embraced a new terminology that includes such terms as ‘leaky gut’, microbiome, SIBO (small intestine bacterial overgrowth), PPIs (protein pump inhibitors), FMT (faecal microbial transplant), FODMAPs (fermentable oligo-, di-, mono-saccharides and polyols), MCAS (mast cell activation syndrome), exclusion diets to eliminate phytochemicals such as histamines, salicylates, oxalates, lectins, etc. As with any innovation, there can be initial confusion, especially when the science has yet to catch up with the interventions already being implemented. In this context, gut health is no exception. It should be noted, too, that some of the essential oils commonly utilised for their perceived ‘natural’ antimicrobial properties against pathogens appear to exacerbate symptoms in some patients, such that more research is required to ensure their safety [5].

Given the growing scientific awareness that the gut–organ axis can underpin many different diseases, this hypothesis includes consideration that the effects of SFN in the gut may also have significant and beneficial effects on distant cell types, organs and systems, thereby serving to potentially prevent and treat both acute and chronic disease [6].

## 2. Background to the Hypothesis—The Case for Sulforaphane

This article catalogues the evolution of and rationale for a hypothesis that seeks to explain how a single low-molecular-weight, highly bioavailable aliphatic molecule, sulforaphane, can contribute more broadly to human physiology by initially targeting the gut epithelium. In doing so, we explore its potential applications in a clinical setting, in which clinicians practising nutritional medicine describe the outcomes of a range of patient cases. Notably, the safety of broccoli sprouts has been established for almost two decades [7].

Of significance is the fact that in many cases where the primary presenting symptoms are related to aberrant intestinal function, complete or partial resolution also occurred in seemingly unrelated conditions such as inflammatory skin diseases, multiple food intolerances, histamine-like allergic reactions, and neuro-psychological disorders. It should be noted that although SFN was the primary and initial intervention, clinicians recommended that their patients consume a mixed diet of minimally processed foods rich in vegetables and other sources of phytochemicals. It was also clear that the dietary recommendations alone were not capable of making the changes that occurred when SFN was added.

The hypothesis was formulated on the basis of the author’s initial clinical observations, revealing that in some individuals, ingestion of even small amounts of an SFN-yielding dried broccoli sprout supplement resulted in marked gastrointestinal symptoms that included bloating, cramping, flatulence and diarrhoea; these symptoms typically subsided with cessation of the supplement, and could potentially clear entirely with titrated re-introduction of progressively increasing amounts up to a typical daily dosage, as reflected in previously published clinical trials [8]. This response may have been the result of the die-off of non-commensal microbiota existing within a dysbiotic gut ecosystem; the hypothesis includes this consideration.

### The Hypothesis

The known fact that the highly bioavailable and potent Nrf2-activating molecule SFN exhibits multiple effects within human cells, including the gut ecosystem and its underlying immune network, leads us to hypothesise that its observed clinically trialled systemic effects may provide significant therapeutic potential across a range of gut–organ axes [9].

## 3. The Emerging Role of the Gut Microbiome in Human Health

Following improvements in DNA-sequencing technologies, the Human Microbiome Project (HMP) was a logical extension of the Human Genome Project (HGP), the latter being completed in the early 2000s. Both projects have provided insights not previously available, and with researchers eager to explore both domains, neither project provided exactly what had been anticipated in relation to being able to better target the aetiology of specific diseases [10].

Clinicians in nutritional medicine rapidly adopted both genetic testing and stool microbiome analysis as soon as they were commercially available and affordable for patients. The recent availability of gene-based technologies for identifying the microbes within us has seen an accelerated drive, aiming to enhance digestive health by clinically manipulating the resident microbial species, especially those of the human intestine; clinical recommendation of probiotics, prebiotics, L-glutamine and antimicrobials have led the charge [11], leading this author to question whether this approach is yielding more questions than answers. Given the emerging protective role of commensal microbes in defence against respiratory pathogens [12] and the fact that the antibiotics used to attack pathogens are collaterally destructive to commensal microbes [13], is restoration of gut homeostasis using either pharmaceutical or phytochemical-derived antimicrobials [14] a practical therapy? One must surely query whether it is even possible to successfully micromanage the intricate and complex relationships of the host and its companion microbial population [15].

### 3.1. The Growing Issue of Food Intolerance

Patients whose digestive tracts adversely react to numerous foods may be encouraged to eliminate entire food families from their diets, such as those that naturally contain phytochemicals like histamines [16], lectins [17], salicylates [18], FODMAPs [19] and others. They may initially feel better for excluding these foods, even though it is unlikely that prolonged elimination explains why the food is reactive in that individual. The removal of dietary lectins (otherwise known as ‘anti-nutrients’) has raised concerns about the possibility that their widespread removal may become the ‘next food fashion’. Proponents of lectin removal encourage their supporters to avoid all plant foods, even claiming that vegetables and other plant foods are toxic for humans [17].

Widely distributed throughout the plant kingdom, lectins are most abundant in legumes and grains. Even though their toxicity when uncooked is well known, soaking, cooking and fermentation irreversibly denature lectins, although notably, microwaving does not [20]. Seldom is there any consideration that the initial improvement achieved by removing most plant foods may lead to marked nutrient deficiencies, a situation arguably capable of impeding recovery and promoting other nutrient deficiency disorders over time. In their whole and cooked forms, there is currently no strong evidence that dietary lectins consistently cause inflammation, intestinal permeability, or nutrient absorption issues in the general population [20]. A study that tested 500 individuals for anti-lectin antibodies found some immunoreactivity in 7.8% to 18% against different lectins, illustrating that some individuals may need to be cautious [21].

In a similar fashion, many of those with IBS or other uncategorised digestive dysfunctions studiously avoid histamine-containing foods in the belief that their symptoms are due to histamine intolerance. Histamine-containing foods can be readily identified online, and represent a long list of foods, whereby the exclusion of such foods over a long period may in turn be predictive of dietary deficiencies [22]. Oxalates are similarly avoided by some in the hope that their removal will relieve their symptoms. Little reported is the fact that oxalate is produced endogenously and hepatic oxalate biosynthesis can contribute 50–80% of the total body oxalate levels [23]. It is little wonder that misinformed individuals attempting to eliminate entire groups of phytochemicals such as histamines, lectins and oxalates find themselves selecting from a very restricted list of foods, typically without enhancement to their overall state of health and wellbeing.

This hypothesis considers that the intolerances to commonly consumed plant foods experienced by some individuals may in fact represent a generalised state of impaired homeostasis at the gut–immune interface, manifesting as microbial dysbiosis and the gut epithelium being in an inflammatory state. Evidence also exists suggesting that IgE-mediated food allergies can result from interactions between the intestinal epithelium and the microbiota [24]. As described below, appropriately dosed SFN has been clinically demonstrated to eliminate IBS symptoms, a finding that may possibly indicate that intestinal homeostasis has been restored, thereby mitigating the symptoms of both food allergy and food intolerance.

### 3.2. Lessons from Nature’s Inbuilt Cellular Mechanisms

In our seemingly insatiable quest to manipulate the composition of the gut microbiome for the enhancement of human health, it is worth contemplating that nature has sustained human life on this planet for millennia—and all without any of the benefits offered by modern technology. Clearly, there are processes embedded within human cells that have allowed them to adapt to their ever-changing environments [25]. With a better understanding of these endogenous mechanisms, it may be possible to formulate clinical strategies that resemble those used by nature herself. It could be that an important piece of the gut-health puzzle has been overlooked, and that a greater focus on restoring the function of the remarkable intestinal epithelial cell is needed in order to redress the ecological balance.

Examination of the endogenous mechanisms of the intestinal epithelium reveals that these cells possess several unique properties [26]. A key element of this hypothesis is to consider whether these properties can be clinically harnessed, thereby providing clinicians with access to a therapeutic strategy capable of restoring homeostasis to the gut ecosystem. Such an approach may therefore provide a clinical strategy that obviates the need to utilise non-selective antimicrobials, be they pharmaceutical or nutraceutical. SFN is a key factor in the initial steps of this therapeutic intervention.

## 4. The Evolution of the Hypothesis

Following research that led to the publication of three review papers on SFN [8,27,28], the author was led to explore its potential in a clinical environment. Over several years, our group observed many favourable clinical outcomes when SFN was employed in the context of an appropriate diet. These cases cover a broad range of conditions across all physiological systems. Section 9 describes several conditions, among which are three dermatological cases, for which photographic evidence of change is provided. These patients had presented with comorbidities, some of which resolved under treatment with SFN in conjunction with dietary and lifestyle advice.

### 4.1. The Evolution of Strategies for Addressing the Unanswered Questions

In considering the available therapies and the possibility that a somewhat different approach may more comprehensively optimise the function of the gut ecosystem, several questions bear consideration. The eight questions that do not yet have satisfactory answers are listed below as a series of dilemmas to be pondered.

Dilemma # 1. If diet alone can dramatically shift the composition of the microbiome within 24 h, what do we expect of a probiotic supplement [29]?

Dilemma # 2. Even though probiotics as food or supplements demonstrate favourable clinical outcomes, they typically do not colonise the gut [30]. Therefore, how do we expect them to restore the diversity and lost species to the gut microbiome after oral antibiotic use [31]? If no trace of an administered probiotic organism can be found a few weeks later, is there any sustained benefit [32]?

Dilemma # 3. The presence of obesity and other diseases is indirectly proportional to the diversity of the microbial organisms inhabiting the human gut. Therefore, what can we expect of a few selected probiotic strains in helping to solve the issue of limited diversity [33]?

Dilemma # 4. There is no accepted antimicrobial approach that selectively destroys a pathogen without, to some degree, impacting the commensals. If we select a tool to ‘kill’ gut pathogens, pathobionts or rogue commensals, how do we avoid damaging the protective commensals with which we live symbiotically [14]?

Dilemma # 5. The value of using a probiotic supplement after antibiotic therapy to recolonise the gut is uncertain. A 2018 multi-centre study showed that probiotic supplementation after treatment with antibiotics delayed gut microbiome reconstitution by around five months [34].

Dilemma # 6. If the gut can harbour around 1000 different species, why do we expect a probiotic supplement harbouring just a few species to favourably modify a human microbiome [30]?

Dilemma # 7. If Lactobacilli make up <0.1% of the total microbes in the human microbiome, why do we so readily choose them as probiotic supplements [35]?

Dilemma # 8. If L-glutamine is a preferred energy source for the small intestine and not the colon, why is it used almost universally in gut repair programmes regardless of the affected region [36]?

This author’s inability to satisfactorily reconcile these issues in the context of a therapeutic strategy addressing gut health is part of the impetus that led to the development of an alternative approach targeting the intestinal cells as the primary focus; in other words, these dilemmas serves as the origin of the hypothesis.

### 4.2. Shifting the Emphasis from the Microbe towards the Host

The development of a healthy gut mucosa is a bi-directional event between the host and the gut microbiota, creating an environment that allows the specific members to establish persistent colonisation via the utilisation of host-derived dietary glycans [37].

A 2018 scientific review entitled Colonocyte metabolism shapes the gut microbiota [38] supported the claim that it is primarily the host colonocyte that drives the microbiome, rather than the reverse. Its authors, Litvak et al., stated that “Because the human immune system already has mechanisms to balance the colonic microbiota, harnessing this host control mechanism for therapeutic means could provide an alternative to targeting the microbes themselves for remediation of dysbiosis”. Although Litvak et al. were focused on the colonocyte, the epithelial cells that exist as a single layer from the mouth to the anus are equipped with a wide range of region-specific processes for restoring and maintaining homeostasis [39].

The endogenous intestinal epithelial cell (IEC) mechanisms include but are not limited to the synthesis of protective mucus by specialised goblet cells, the synthesis and release of sIgA by plasma cells, the production of selective antimicrobial peptides by Paneth cells, and the synthesis and release of several hormones by the Enteroendocrine Cells. In addition, IECs contain sophisticated monitoring systems that include Toll-like receptors and dendritic cells to detect possible threats to which healthy IECs can respond [40].

Where the popular current focus on addressing dysbiosis is on manipulating the microbiota through the application of antimicrobials and pro- and prebiotics, it may be time to shift the emphasis closer to optimising colonocyte metabolism as the primary driver of dysbiosis in the colon. Since these mechanisms within the human gut ecosystem already exist, the author of this hypothesis suggests that it may be advantageous to intervene at this level, as distinct from using antimicrobials and exogenous probiotic strains to influence host cell function.

It is here that SFN, as a naturally occurring food molecule, becomes relevant as an intervention. SFN is both potent in its ability to upregulate the expression of a battery of cytoprotective genes and is also highly bioavailable compared to the more abundant food-derived polyphenols [26,27]. As detailed later, SFN exhibits several functions capable of influencing the gut ecosystem in the direction of homeostasis.

By highlighting the role of IECs as the ‘Mission Control’ of the gut ecosystem, this article proposes an alternate therapeutic strategy directed at optimising the processes nature has used for the millennia for which human life has existed. The proposed approach, underpinned by the hypothesis presented in this article, targets the host IECs as its initial and primary focus in the restoration of luminal microbial composition, rather than expecting a secondary host response via an introduced probiotic strain.

## 5. Focusing on Sulforaphane’s Clinically Relevant Properties

The body of literature on glucosinolates and their enzymatic degradation products, the isothiocyanates, has been rapidly growing over the past three decades. Where the initial publications focused on their roles in the plant kingdom, recent years have seen an explosion of interest in their potential roles in human health. Of all dietary vegetables, crucifers are considered to be the most capable of conferring significant benefits on human health [41], with early studies linking this plant family to cancer prevention [42]. More recently, their clinical applications have expanded, with positive clinical outcomes having been reported in seemingly unrelated conditions [43,44].

### 5.1. Unravelling the Mechanisms of Action

The early 1990s saw the first of a series of research publications exploring germinated broccoli seeds (*Brassica oleracea italica*) as a significant source of the isothiocyanate SFN [45], which has been shown to be far more abundant in young sprouted seeds than in the mature broccoli vegetable [46]. Although the mechanism was not initially known, bioactive SFN was shown to be a potent inducer of the phase II detoxifying enzymes quinone reductase (NAD(P)H: quinone oxidoreductase 1 (NQO1)) and glutathione-S-transferase (GST). Two years later, the transcription factor Nrf2 (encoded by the *NFE2L2* gene) was isolated [47], and its activation by SFN was later shown to be essential in the induction of phase II enzymes, together with around 250 other cytoprotective genes that are transcriptionally regulated in this way [48].

### 5.2. Nutrigenomics in Action—Enter Nrf2

The finding that an entire bank of cytoprotective genes can be induced via a single transcription factor Nrf2, that is in turn activated by bioactive food-derived molecules, has significant implications for human health. Emerging evidence shows that Nrf2 lies at the centre of a complex regulatory network and establishes it as a truly pleiotropic transcription factor. Its activity is tightly regulated through a complex transcriptional and post-translational network that enables it to orchestrate the cell’s response and adaptation to various pathological stressors for the maintenance of homeostasis [49].

The ensuing thirty years has seen almost 3000 indexed scientific publications on SFN. Over seventy of these report clinical trials, most of which demonstrate positive outcomes across a diverse range of common health abnormalities which include asthma, emphysema, nasal allergy, autism, Type 2 diabetes and *Helicobacter pylori* gastric infection. Of clinical significance is that in each case, the quantity of SFN administered in the trials can be achieved using practical daily doses of fresh broccoli sprouts or a dried broccoli sprout supplement standardised for an approximately equivalent SFN yield [8,43].

### 5.3. Nrf2 and the Concept of Upstream Effects

A seminal 2018 drug discovery paper showed that Nrf2 activation induced positive responses in metabolic, inflammatory and autoimmune disorders, as well as in diseases of the lung, liver, kidney, gastrointestinal (GI) tract, cardiovascular system and neurological conditions [50]. The authors described the role of Nrf2 in human chronic disease from a systems medicine perspective, referring to a map of altered Nrf2 disease mechanisms as the Nrf2-diseasome and a separate map of Nrf2 with other physically or functionally associated proteins as the Nrf2-interactome.

What soon becomes apparent with such diversity of response to Nrf2 activation is [51] that common upstream cellular processes must be at play; furthermore, these effects are achievable using a practical daily SFN dose of between 20 and 40 mg [8]. Although laboratory researchers typically record such doses as micromoles, milligram weights are shown here in keeping with the data provided by clinicians in their case reports.

As discussed later, the nature of the food-derived molecule SFN and its diverse documented applications is the mechanistic foundation on which this hypothesis sits. Although clinical trial data utilising SFN in human intestinal conditions are scant, we go on to describe observational findings that can be linked to a series of relevant published in vitro studies.

### 5.4. SFN as a Nature-Compatible Strategy for Harnessing the Power of Nutrigenomics

We posit that to enhance the function of IECs as a first step in restoring the gut ecosystem, a novel nutrigenomic approach to targeting the core upstream factors governing cellular defences could be employed. Phytonutrients, including SFN, that potently activate these core processes have been identified and are sufficiently bioavailable to achieve this end [52]. As the gut functions with the continuous challenge of responding to pathogens while remaining relatively unresponsive to commensal microflora, food proteins and other antigens, restoring homeostasis to IECs can be readily justified as a key initial step [53].

### 5.5. Sulforaphane—A Potent Multifunctional Phytonutrient

Nutrigenomically active SFN is a potent inducer of hundreds of genes associated with cellular defence mechanisms. In this context, these core upstream genes include those that code for antioxidant and phase II detoxification enzymes, the antioxidant glutathione, and the heavy metal chelator metallothionein [54]. In addition to activation of Nrf2, SFN effectively downregulates Nf-kB, a transcription factor that promotes inflammation; both transcription factors exhibit cross-talk effects that collectively enhance cytoprotection and inhibit uncontrolled inflammation. SFN favourably influences both [55].

### 5.6. Collaborative Contributions of SFN and the Microbiota to Gut Homeostasis

The cell walls of Gram-negative bacteria increase gut luminal levels of lipopolysaccharides (LPS), which are detected by and bind to Toll-like receptor 4 (TLR4). This initiates the activation of Nf-κB with the subsequent generation of inflammatory cytokines that are systemically absorbed [56]. At least three apparently distinct mechanisms—endoplasmic reticulum stress, Toll-like receptor (TLR) 4 activation, and changes in gut microbiota—have been identified as triggers of obesity-associated metabolic inflammation [57]. SFN has been identified as a molecule that can reduce inflammation via inhibition of LPS-TLR4 binding [58,59]. These processes may, in part, explain beneficial effects documented to be exerted by SFN on metabolic imbalances, including Type 2 diabetes [51].

**Figure 1:**
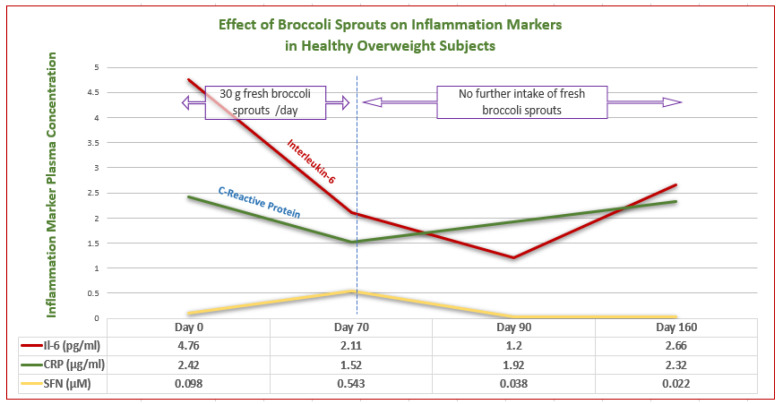
A clinical trial using 30 g of fresh SFN-yielding broccoli sprouts daily as the intervention over 70 days showed that the markers of inflammation Interleukin-6 (IL-6) and C-reactive protein (CRP) were downregulated for the 70 days during which the sprouts were consumed. After cessation of treatment with the sprouts, IL-6 trended lower until measured again at 90 days. By contrast, when sprout intake ceased at 70 days, CRP again increased, so that when measured at 90 days, it had increased, although at that stage, it had not yet reached the baseline level. (Graph created from data from Lopez-Chillon et al. [65]).

More specific gut and immune-related effects include the inhibition of bacterial urease in the control of *Helicobacter pylori* infection [60], induction of endogenous antimicrobials such as beta-defensin by the Paneth cells [61], inhibition of LPS-endotoxin binding to Toll-like receptor 4 (TLR4) [62] and inhibition of Substance P to limit mast cell histamine [63,64]. In addition, SFN has been shown to potently downregulate, via Nf-kB, the inflammatory cytokines IL-6 and C-reactive protein (CRP) in overweight, otherwise healthy adults, with these effects being sustained over an extended period [65]. Figure 1 illustrates these properties.

A further benefit of this molecule is that, unlike polyphenols which exhibit very low bioavailability of around 1–10% [27], in a rodent model, SFN was shown to exhibit an absolute bioavailability of around 80% [66].

### 5.7. The Role of Sulforaphane in Cellular Defence Mechanisms

In the last 25 years, the mechanisms used by cells to defend themselves against a variety of threats to their integrity have become much better understood. As the science has evolved, it has become clear that the free radical–antioxidant theory of the past was just too simplistic, and that high doses of direct-acting antioxidant vitamins, in particular, can inhibit the cell’s protective responses by masking nutrigenomic signals [67]. This is believed to be because the signals that cells use to upregulate their own defences are stressors, to which the cell responds by upregulating its endogenous defences. If an exogenous antioxidant source greater than can be practically ingested via the diet that artificially skews the cellular redox balance, the effect of a pro-oxidant stressor may be masked; as a result, the cell will not ‘realise’ that it should respond by upregulating the appropriately cytoprotective genes.

Of note is the fact that this principle has not yet gained wide acceptance by clinicians, who typically recommend supraphysiological doses of direct-acting vitamins such as vitamins A, C, E and Beta-carotene, together with N-acetyl-cysteine (NAC), the latter having become very popular as a glutathione precursor. NAC is not without adverse effects, some of which impact normal intestinal function. However, it is little known that histamine secretion can be induced by NAC, which is thought to be due to a direct secretagogue effect of the drug on mast cells and basophils; asthma is known to be exacerbated by NAC in some asthmatics [68]. 

With the potential to compound the effects of elevated histamine in susceptible individuals, NAC has also been shown to inhibit the activity of diamine oxidase (DAO), the primary enzyme in the catabolism of biogenic amines (including histamine) in the intestine; even 30% inhibition is considered to be a critical level [69].

### 5.8. Harnessing-Compatible Cellular Defence Mechanisms

The aforementioned properties highlight two potential issues when using NAC as a therapeutic intervention in patients with intestinal conditions: (1) NAC may exacerbate histamine-related symptoms; and (2) NAC and other supplemented direct-acting antioxidants may inhibit the induction of endogenous cytoprotective genes via Nrf2, described as “a master redox switch in turning on the cellular signalling involved in the induction of cytoprotective genes” [52].

By deduction, it would seem that nature can potently activate Nrf2 when an individual consumes large quantities of plant foods, especially non-starchy varieties. A 2010 clinical trial by Hermsdorff at al. showed that biomarkers of inflammation, CRP, TNF-alpha, IL-6 and others, together with homocysteine, were significantly lowered by a diet containing >660 g of (non-organic) vegetables daily [70]. It is likely that the same or greater quantities of vegetables simultaneously upregulated Nrf2 and/or downregulated Nf-kB, two key transcription factors shown to act in concert [55,71].

It is especially important that this hypothesis, which considers the diverse upstream properties of SFN in the function of human cells in general, must encompass the function of the gut barrier as an integral part of the gut ecosystem. The influence of SFN in key aspects of the gut barrier will become apparent as the discussion unfolds.

For the clinician, it is useful to realise that the most potent known single-food-derived activator of Nrf2, SFN, is capable of upregulating the protective genes in human cells, including enterocytes and colonocytes [72]. Piotrowska et al. described mechanistic links to Nrf2 throughout the entire digestive tract, including its role in maintaining the gut barrier. They also stated that currently used drugs that modulate Nrf2/Keap1 may be effective in the treatment of IBD. In an era in which it may not be possible to persuade patients to consume >660 g of vegetables daily, a high-SFN-yielding whole broccoli sprout supplement may be an appropriate prescription [27].

## 6. The Gut Barrier

The gut barrier is defined by a single layer of IECs, which act as the boundary separating the body from its external environment, the latter represented by the gut lumen [73]. It performs a pivotal role as the first physical barrier against external factors, and maintains a symbiotic relationship with commensal bacteria. This barrier allows the passage of water, food-derived nutrients, and a selection of microbe-derived molecules through to the underlying cellular network, with the simultaneous goal of excluding potentially toxic microbes and molecules. The latter can be transported from the luminal to the apical side of the epithelium by both trans- and paracellular routes, based on selectivity with respect to both size and charge [74].

### 6.1. Tight Junctions as Critical Components of the Gut Barrier

Paracellular junctions connect IECs to each other, with tight junctions (TJ) separating each cell from its neighbours. TJs are an essential component of a normally functioning intestine [74], and form a complex mechanism that is somewhat analogous to a spring-loaded hinged gate with several types of latches that allow it to ‘open’ and ‘close’. Specialised environment-responsive cellular proteins—occludins, claudins and junctional adhesion molecule (JAM)—act as the latches, with zonulin acting as the spring. These epithelial proteins are attached to intracellular actin and myosin filaments, allowing the TJ to exist in a dynamic state in which the relaxation or contraction of the TJ allows the entry or exclusion of microbes or large molecules, as appropriate [75]. When this mechanism is perturbed, the gut barrier is compromised, and intestinal permeability is increased, allowing the entry of unwanted molecules and/or microbes.

The term ‘leaky gut’ has crept into the popular vernacular but misrepresents the dynamic nature of the gut barrier; consequently, interventions to address the issue clinically are often complicated by the fact that laypeople believe that the gut barrier is akin to a pipe in which there are holes that must be ‘sealed’. More correctly, the components of the TJ respond continuously to their immediate environment, ‘opening’ and ‘closing’ upon the appropriate biochemical cues.

### 6.2. Exogenous Factors Impacting the Tight Junctions

**Figure 2:**
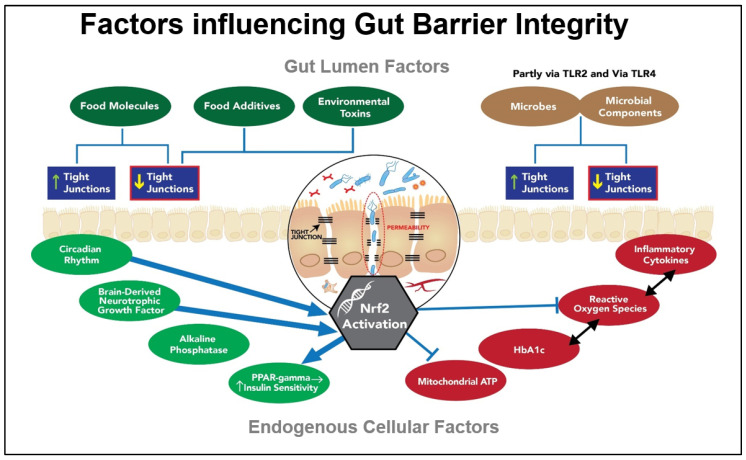
Key factors contributing to gut barrier integrity. Through its TJs, the gut barrier is impacted by numerous exogenous factors, including food, environmental toxins, microbes, and their metabolites. It is more significantly impacted by endogenous factors associated with inflammation, oxidative stress, and poor metabolic control. Hyperglycaemia is directly correlated with poor gut barrier function [82].

Both dietary factors and endogenous metabolic factors [76] provide signals that are known to influence the components of the TJs. Gluten research has confirmed its role in destabilising zonulin, and thereby in relaxing the TJs; however, gluten is just one factor of many [77]. Gluten is widely considered within the lay community to be the primary dietary factor responsible for destabilising the gut barrier. This notion, together with that promoted in a widely publicized book [78] claiming an association between wheat intake and adiposity, has led to the growing trend of gluten avoidance behaviour in many countries, even when neither coeliac disease nor gluten intolerance is present [79,80]. Figure 2 illustrates both exogenous and endogenous factors contributing to the dynamics of the gut barrier. The feed-forward loop between gut barrier dysfunction and glucose dysregulation is a theme advanced by other researchers investigating the role of the gut barrier in a range of systemic disorders [81].

Although gluten’s effect on destabilising the TJs is acknowledged, it is less well known that there are many food molecules that impact the TJs. Among the other food molecules that tend to relax or open the TJs are alcohol, piperine, capsaicin, and hops, together with capric and lauric fatty acids from coconut oil. By contrast, some of the foods that tend to tighten the junctions include phytochemical flavonoids, long-chain omega-3 oils, fucoidan, glutamine, and SFN, as well as the nutrients vitamin A, vitamin D, and zinc [83].

In addition, certain probiotic strains, as well as microbial degradative products of prebiotic metabolism, such as butyrate, can beneficially influence the TJs that separate the IECs [77,84]. It is noteworthy that many of the additives and processing aids being used for the commercial production of gluten-free foods with the appearance, taste and mouth-feel of the gluten-containing original have been shown to adversely affect gut-barrier integrity [85]. Food industry processing aids such as the enzyme transglutaminase, together with several commonly used emulsifiers, have been identified to exhibit destabilising effects on the gut barrier, which in some cases are thought to contribute to a rise in autoimmune conditions [86].

According to a recent paper, there are no FDA-approved therapies that can be used in clinical practice that are capable of recovering the epithelial tight junction barrier [87], a fact that would support the value of this hypothesis if its fundamental premise as a nature-compatible strategy can be validated.

Of significance in the context of our hypothesis is the fact that zonulin, occludin and the claudins are influenced by Nrf2 activation [72]. This hypothesis proposes that the ability of SFN to induce the genes coding for these proteins via Nrf2 activation could help to restore their normal function, presumably supporting SFN’s effect on uncontrolled inflammation, known to disrupt the gut barrier [88].

### 6.3. Endogenous Factors Impacting the Gut Barrier and Beyond

It now appears that the removal of gluten and the administration of probiotics have a lesser impact than endogenous factors such as the elevated glycated haemoglobin (HbA1c) typical of diabetic individuals. Perhaps surprisingly, it has recently been discovered that one of the most significant factors driving gut barrier dysfunction is hyperglycaemia. In a 2018 study entitled Hyperglycaemia Drives Intestinal Barrier Dysfunction and Risk for Enteric Infection, the authors described glucose as “an orchestrator of intestinal barrier function” [81].

**Figure 3:**
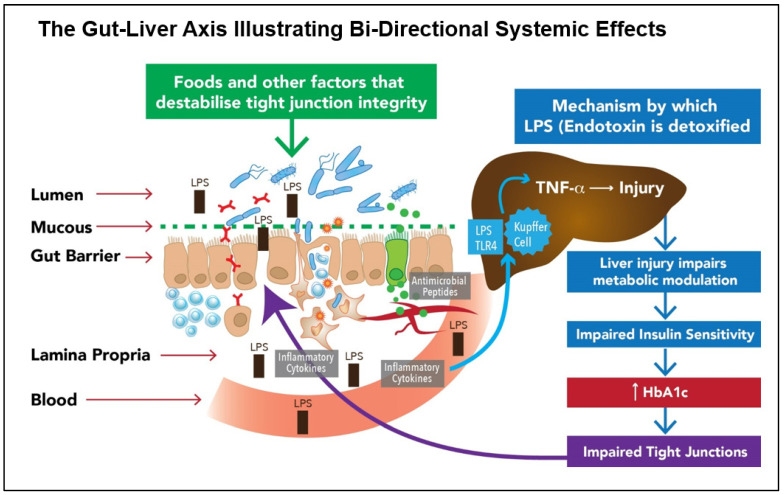
The intricate bidirectional mechanisms between the gut ecosystem and systemic organs as the primary driver of chronic disease. Gut barrier dysfunction and glucose dysregulation drive metabolic disease in a self-perpetuating loop. When the gut barrier is impaired, LPS has two primary effects: (1) entry via the paracellular spaces to the bloodstream and (2) initiation of the synthesis of inflammatory cytokines. LPS travels via the bloodstream to the liver, where it attaches to TLR4 to initiate inflammation with subsequent hepatic damage. Such damage leads to insulin resistance and elevated HbA1c. In turn, HbA1c further impacts the gut barrier, further contributing to an influx of LPS and antigens. (Image adapted by the author from Kirpich IA et al. [56]).

The researchers, Thaiss et al., showed that hyperglycaemia reprograms IECs and that the genes most affected are associated with tight junction modulation. They also show that TLR4 ligands such as endotoxin-LPS are directly correlated with HbA1c [81]. The significant contribution made by the gut bacteria to non-alcoholic fatty liver disease (NAFLD) is multifactorial, with small-intestinal bacterial overgrowth (SIBO) having been linked to the development of fatty liver disease. Figure 3 illustrates the bi-directional loop, integral to the gut–liver axis, linking an impaired gut barrier to glucose dysregulation and which may hold the key to the clinical management of both disorders.

## 7. Restoring Homeostasis to the Gut Ecosystem

Tolerogenic IECs naturally recognize and interact with commensal bacteria and give instructions to the underlying mucosal immune cells to “initiate an immunological balance between active and quiescent conditions, eventually establishing intestinal homeostasis” [40]. It is here that the sciences of cell biology, microbiology and immunology intersect, highlighting the complexity necessary for the gut ecosystem to remain in homeostasis.

Although once regarded as unwelcome ‘germs’, our commensal microbial ‘companions’ are now considered essential for human health. As the science continues to unfold, it is becoming clear that intricate signalling and cross-talk takes place between these microbes and their human hosts, which is joining the dots in our understanding of why the presence of a diverse microbiota benefits not just digestive health but assists in driving the processes of health or disease in distant organs [89].

Although it is tempting to consider that probiotics might achieve the desired response, clearly, this would address only one-half of the bi-directional relationship between the host and its resident microbiota, notwithstanding the fact the eight largely unanswered questions raised earlier in Section 4.1 remain.

### 7.1. Probiotics—Longstanding Therapy or Recent Innovation?

In the history of human life on this planet, probiotic supplementation might be considered a relatively recent therapeutic intervention. However, prior to refrigeration as a means of food preservation, it is certain that substantial amounts of a diverse array of food-derived bacteria, yeasts and other micro-organisms were ingested daily by our human ancestors. Modern science continues to demonstrate the value of cultured foods in human health in relation to their known impact on the gut–immune interface [90,91].

In this way, humans have been exposed to a large variety of microbes, which have most certainly been ingested daily from the broader environment, as well as from soil and food, whether intentionally fermented or not. Due to the combination of refrigerated storage and an obsessive need to eliminate exposure to microbes in the name of hygiene and food preservation, the human microbiome in industrialised populations is demonstrably very different from that in those still adhering more closely to their traditional lifestyles. This modern approach to hygiene has been mechanistically linked to the rise in the manifestation of food intolerances and allergic diseases, especially in children [92].

### 7.2. Symbiosis between the Host and Luminal Microbes

The host’s diet supplies food, which contributes to the nutrition of the microbiota, while the microbes in turn release vitamins and metabolites, including some that attach to epithelial receptors, from where they act as signalling molecules, essential in a range of host functions [93,94]. The host and its microbiome exist in a synergistic relationship that is an essential component of gut homeostasis [95]. Several families of Toll-like receptors located on the surface of the gut epithelia form part of an elaborate signalling system that is too complex to detail here, although it has been extensively covered in other publications, two of which are cited [96,97]. Nevertheless, the dietary guidance used to support the role of SFN in cases based on this hypothesis utilise these principles in the form of recommendations to regularly consume cultured foods as rich sources of *Lactobacilli* spp. [98]. In clinical management, these and other gram-positive microbes, such as those naturally found in sauerkraut, kimchi, yoghurt, kefir and other fermented foods, are introduced only after noticeable reactivity to particular foods has diminished, especially since fermented foods may also release histamines. This author’s clinical observations suggest that many of those who report significant intolerance to a range of foods find that their intolerance extends to fermented foods, with many claiming to be reactive as well to probiotic supplements, a dilemma confronted by those clinicians who rely on the recommendation of probiotic supplements as an integral part of their ‘gut therapy’.

## 8. Determining an Effective Gut Repair Strategy

A recent 2019 Australian survey indicated that most complementary medicine clinicians addressing gut repair use a range of interventions that typically include multi-strain probiotics (including *Saccharomyces boulardii)*, L-glutamine, zinc, vitamin D and curcumin, together with the elimination of gluten and alcohol [11]. Others have also reported that strain-specific probiotics are frequently a mainstay of treatment for patients with digestive issues [99].

A recent review investigated the effects of probiotic supplementation in healthy adults, showing that, although such supplementation can lead to transient improvement in the concentration of the specifically supplemented bacteria in the gut microbiota, providing benefits in several conditions while present, it fails to support the ability of probiotics to cause persistent changes in gut microbiota [100].

Individual probiotic strains available as supplements have been well researched for a range of effects that are beneficial to the host while ‘in residence’, including modulation of immune function, production of organic acids and antimicrobial compounds, interaction with resident microbiota, improvement of gut barrier integrity, and synthesis of vitamins. However, they are not without their risks, especially in patients who are immunologically compromised [101]. Furthermore, whereas it was once thought that the purpose of probiotics was to ‘crowd out’ less desirable microbes through competitive exclusion, it is now known that signalling from the microbe to the IEC results in a far more nuanced effect. Moreover, this occurs whether the probiotic organism is alive or dead [102,103,104], a finding that tends to support the notion that probiotic organisms may deliver their benefits via signalling molecules on their membrane surface.

Gram-positive commensal microbes such as *Lactobacilli* spp. can be identified because they carry a specific molecule on their outer cell walls. Lipoteichoic acid (LTA) acts as a ligand for Toll-like receptor 2 (TLR2), and when LTA binds to TLR2, this initiates an extremely complex set of immune-signalling responses. These responses include the synthesis of interferons, natural killer cells, and cytotoxic lymphocytes to control infection via Th1 polarisation, simultaneously downregulating Th2, thereby inhibiting an allergic response [105]. As discussed by Johnson et al., this mechanism plays a critical role in gut barrier function. It is possible that the novel approach suggested by this hypothesis, which is designed to mimic the mechanisms of nature in targeting the IEC, may be the missing piece of the gut-health puzzle.

### 8.1. The Potential Impact of Sulforaphane on Restoring Gut Homeostasis

As the most potent naturally occurring Nrf2 activator, SFN also demonstrates an absolute bioavailability of around 80% [66]. As described in Section 5.7, it has been described as “a master redox switch in turning on the cellular signalling involved in the induction of cytoprotective genes”. These properties have relevance to its role in the gut epithelium.

### 8.2. Relevant Mechanisms

In formulating this hypothesis, several mechanisms demanded our consideration. Even though there are limited clinical trial data supporting our hypothesis, we observed significant beneficial effects of oral SFN in helping to normalise gut function; these mechanisms were discussed in previous sections. Moreover, we observed significant benefits in terms of systemic effects, several of which are described and illustrated later.

**Figure 4:**
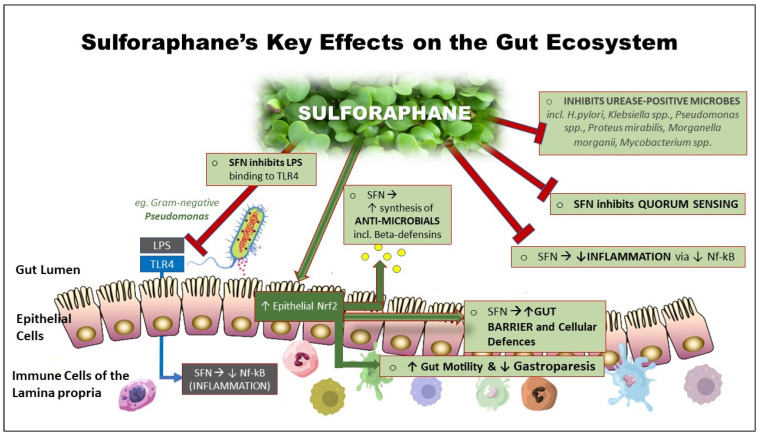
SFN’s key effects on the gut ecosystem: SFN exhibits multiple effects on the gut ecosystem, many of which are associated with its ability to activate Nrf2, which in turn induces the expression of a range of cytoprotective genes.

The putative mechanisms, as illustrated in Figure 4, are as follows:

- SFN INHIBITS GRAM-NEGATIVE BACTERIAL LPS BINDING: SFN inhibits the action of LPS in binding to the epithelial receptor, TLR4, thereby reducing the signalling cascade that leads to the induction of pro-inflammatory mediators via Nf-kB. This is one of several ways in which SFN can downregulate uncontrolled inflammation.
- ENHANCED CYTOPROTECTION: SFN activates epithelial cell Nrf2, thereby inducing around 200 cytoprotective genes; the effects of these include stabilising the gut barrier. Activating the Nrf2 pathway reduces oxidative stress and uncontrolled inflammation while simultaneously downregulating the pro-inflammatory transcription factor NF-kB. In so doing, SFN helps to restore gut immune homeostasis.
- NORMALISED GASTRIC MOTILITY (suppression of gastroparesis): Loss of antioxidant gene expression has been shown to contribute to the development of gastroparesis, leading to Nrf2 being considered to be a potential therapeutic target [106].
- STABLISATION OF GUT BARRIER: SFN may beneficially impact one or more of the endogenous factors that contribute to dysfunctional gut barriers. Of significance are the imbalances in inflammation-redox status and elevated HbA1c.
- SYSTEMIC EFFECTS: Where bacterial die-off may occur in dysbiotic individuals with impaired barrier function, potentially toxic molecules may travel via the portal circulation to the liver, where they must be detoxified. If this process is too rapid, unpleasant systemic symptoms may result. (Reducing the dose and frequency of SFN was observed by the author to ameliorate this effect.)
- ANTIMICROBIAL EFFECT: Of the Nrf2 target genes, the expression of antimicrobial beta-defensin is relevant. Endogenously synthesised antimicrobials that include beta-defensin can selectively target pathobionts or other undesirable microbes without adversely affecting the commensals [107].
- QUORUM SENSING: Biofilm degradation: In vitro studies have shown that SFN can degrade periodontal biofilms that can prevent the resolution of infections, thereby exposing the microbes to attack from elements of both the innate and adaptive immune system. Mucosal biofilm communities are also known to inhabit the human intestinal tract [108], with the potential for SFN to disrupt these biofilms. In so doing, a significant population of microbes is released into the intestinal mucosa, upregulating and potentially overloading detoxification pathways [60,109,110]. We hypothesise that this may partially explain why guided introduction of SFN is important in individuals suspected of harbouring a dysbiotic population of gut microbes.
- UREASE INHIBITION: SFN is a urease inhibitor and has been shown to block the ability of *H. pylori* to produce urease, the enzyme responsible for the development of gastric inflammation and potential gastric tumour development. Many other pathogens/pathobionts are urease positive, including *Klebsiella*, *Staphlococcus aureas*, *E. coli*, *Morganella*, *Pseudomonas*, and many others. *Mycobacteria* (mould) are also urease positive. It is not known whether urease-positive organisms other than *H. pylori* are responsive to SFN [60,110,111].

## 9. Case Studies

Over the 4 years since we began exploring the potential of SFN as a tool for addressing gut health and systemic disease via the gut ecosystem, we have catalogued a range of conditions for which this approach has delivered beneficial outcomes. The author’s 2019 review paper questioned whether SFN had ‘come of age’ as a clinically relevant nutraceutical. Our subsequent implementation of the principles described in that paper, together with our findings of the potential role in addressing both gastrointestinal and systemic disease, provided numerous cases for which conventional treatments had not achieved successful outcomes.

### 9.1. Gastrointestinal Dysfunction with Food Intolerances

CASE # 1—IBS-C: A 38-year-old female presented with a history of severe painful constipation (IBS-C) since childhood. Her comorbidities included chronic migraine that coincided with the start of oral contraceptive use, periodic oesophageal cysts, menorrhagia, fibromyalgia and chronic fatigue. She requested treatment primarily for constipation and intolerance to a range of specific foods. She had aimed to eat a wholefood, pescatarian diet but this was found at consultation to be high in carbohydrates, with her tending to over-eat. She had to learn to avoid numerous foods that tended to exacerbate her symptoms. Some cases of IBS-C are due to a proliferation of a specific pathobiont, *Methanobrevibacter smithii*, a gut microbe that produces excessive methane capable of suppressing the neural activity required for gut peristalsis [112]. However, no data exist to confirm or deny this possibility.

**Figure 5:**
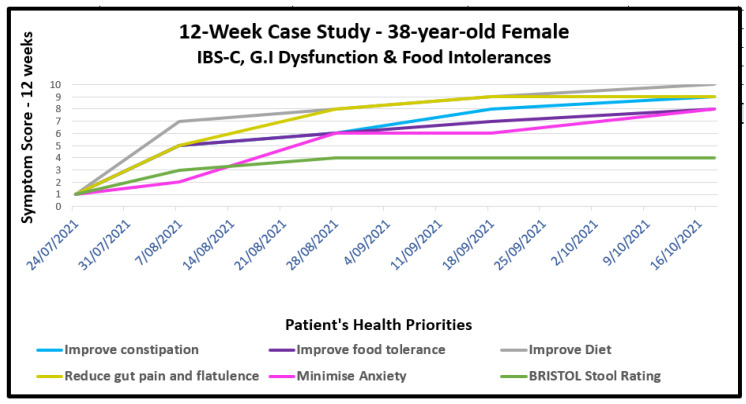
A case study in IBS-C and food intolerances. Tracking the progress of a 38-year-old female whose health priorities resolved to varying degrees, charted subjectively at regular intervals over 12 weeks, using a 10-point Likert scale. The patient was able to completely eliminate all laxatives, on which she had been dependent since childhood. She regained tolerance to foods to which she had been previously reactive.

She was started on an SFN-yielding whole broccoli sprout supplement, titrating the dose to minimise possible exacerbation of discomfort; she was able to tolerate 20 mg daily after several weeks; the dose was increased to 40 mg daily by 6 weeks. At the same time, both her macro- and micro-nutrient balances were improved with dietary guidance that gradually increased non-reactive vegetables. As her inintolerance to previously reactive foods decreased, prebiotic-rich foods were introduced, beginning with supplemental partially hydrolysed guar gum (PHGG), which is known to be generally well-tolerated in children and adults suffering constipation [113]. Heat-killed probiotics, sometimes described as *immunobiotics,* formed part of her clinical recommendations as a way to introduce bacterial wall LTA as a TLR2 ligand, as described in Section 8. By 8 weeks, and after gradual dose reduction, the laxatives Caloxyl and Pariet were no longer required. The progress of her IBS-C and comorbidities is shown visually in Figure 5. Here, the clinician used a 10-point Likert Scale as a guide to the patient’s subjective comments on progress across six parameters associated with her primary health concerns. The scale evaluated responses from 1 to 10, where 10 represented the highest level of wellbeing.

### 9.2. Dermatological Conditions with and without Comorbidities

CASE # 2—PLAQUE PSORIASIS: The first case that alerted the author to the potential of SFN to benefit chronic intractable disease was recorded in 2011. This case was, in some ways, the catalyst for the development of this hypothesis, with the patient responding quickly to SFN as the single new intervention for a case of plaque psoriasis.

The patient presented as a 50-year-old otherwise healthy male consuming a generally good diet and with no negative lifestyle habits; the patient had been regularly taking a multivitamin mineral supplement and a fish oil capsule for some years. He had experienced typical plaque psoriasis for a period of around 20 years, appearing after an episode of severe emotional stress. His elbows and knees regularly bled, causing him considerable workplace embarrassment. Other parts of the body, apart from his eyelids, were easier to conceal.

**Figure 6:**
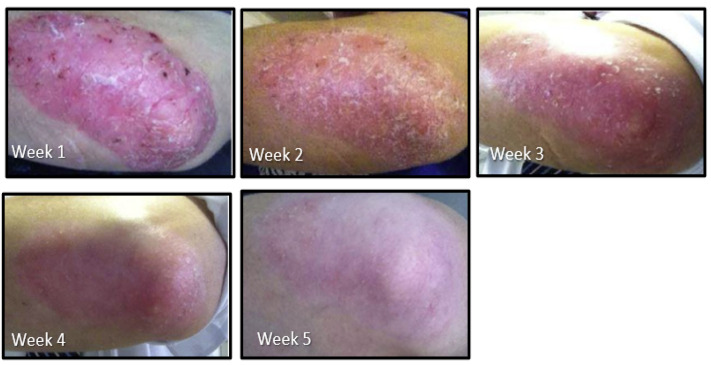
Progress over 4 weeks of the left elbow of a 50-year-old male with chronic plaque psoriasis using an SFN-yielding whole broccoli sprout supplement as the single intervention. (Photographs—November 2011).

Psoriasis is a genetic immune-related hyper-proliferative inflammatory skin condition that affects about 3.1% of the U.S. population [114]; comorbidities are common [115]. It appears in many different forms for which there is no known cure but is typically managed by cycling topical steroids and prescribed oral medicines. The progress of psoriasis on the patient’s elbows over 4 weeks is illustrated in Figure 6. Periodic contact with this man, who is no longer a patient, reveals that his pattern is to take an SFN-yielding myrosinase-active whole broccoli sprout supplement daily for around 3 months (dose ~20 mg SFN daily), by which time the skin clears. However, the plaque gradually develops again over a few months, leading him to resume the supplement when he again feels the need. He started with a dose of 20 mg daily and has never experienced any adverse effects from the SFN-yielding supplement.

**Figure 7:**
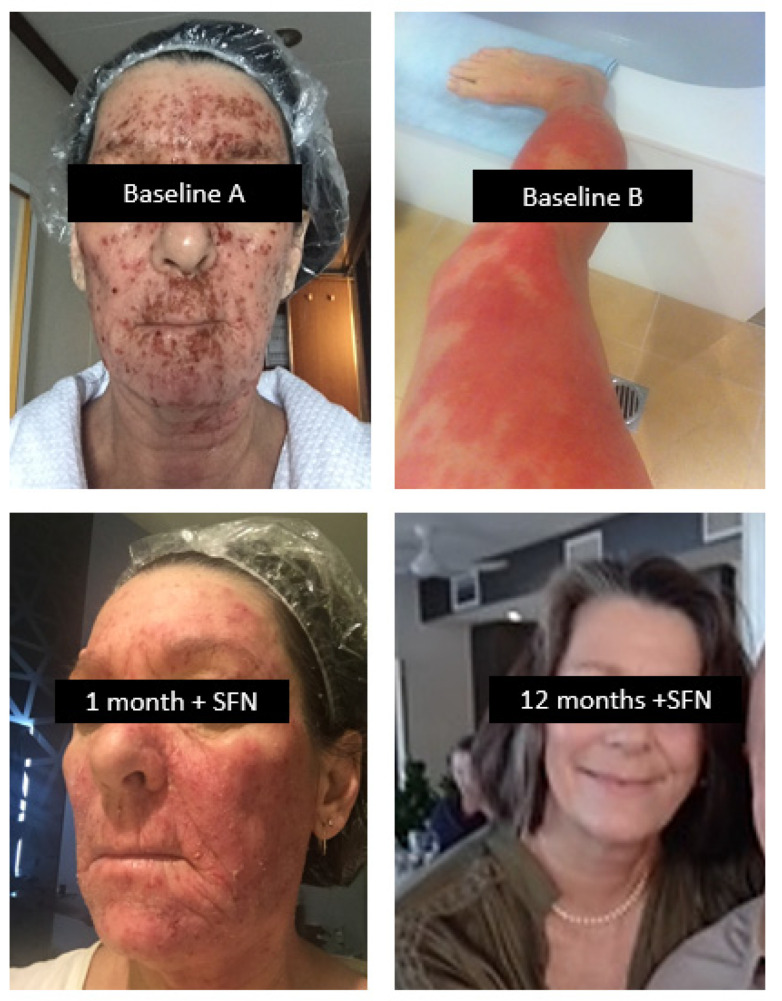
SEVERE CHRONIC ECZEMA. A 40-year history of severe eczema, initially on the hands only but which later spread over most of the body and which worsened after childbirth. SFN, together with an appropriate diet of whole, minimally-processed foods gradually resolved the skin condition, together with comorbid hypothyroidism and hyperlipidaemia. The patient found that tolerance to a wider range of foods accompanied her overall progress.

CASE # 3—ECZEMA: A 62-year-old female neonatal intensive care nurse developed eczema after frequent handwashing at work in 1982. Initially localised to the hands, it rapidly spread to her entire body; her condition significantly worsened after childbirth 9 years after initial onset. She controlled the symptoms with topical steroids. A decade later, she experienced a severe outbreak that became infected, affecting 70% of her body, as shown in Figure 7 as Baseline A (face) and Baseline B (legs). Her skin flared as she became noticeably intolerant to foods that included red meat, all fish, nightshade vegetables, green leafy vegetables, fermented foods, histamine-containing foods, and foods eaten the day following preparation (histamine synthesis increases in stored cooked foods). Medical investigation additionally revealed hypothyroidism, elevated liver enzymes, and elevated LDL cholesterol.

In 2019, she consulted a clinician who recommended an SFN-yielding whole broccoli sprout supplement supplying 20 mg SFN daily together with a diet that strictly removed all foods she knew to be reactive. It was many months before she could add green vegetables to her primarily vegetarian diet. As her tolerance to foods gradually improved and the skin inflammation subsided, the clinician introduced small quantities of the plant foods that had been eliminated as a challenge to her tolerance. As she was able to tolerate SFN, heat-killed probiotics were gradually introduced together with prebiotics as a means of supporting the gut microbiota.

After 12 months of treatment, her skin had fully cleared (SFN + 12 months) and a pathology review revealed normal thyroid and liver function as well as serum lipids. She was able to eat most foods including all green vegetables and fermented foods such as sauerkraut, kefir and kombucha as part of a balanced whole food diet (Figure 7).

CASE # 4 ROSACEA (FACIAL AND OCULAR): An anxious 40-year-old female presented in March 2022 with a history of endometriosis, ovarian cysts and IBS-C. Following laparoscopic surgery in 2018, she experienced a bronchial infection lasting 4 weeks, followed by the onset of facial rosacea. The rosacea was characterised by facial inflammation, redness and flushing, and visible blood vessels on either side of nose, with painful and itchy raised lesions on the forehead, nose, cheeks, chin, ears, and mid chest; her skin was visibly flaking.

The following year, in 2019, she developed amenorrhea. Her own attempts to resolve the rosacea with dietary restrictions appeared to exacerbate the condition. A dermatologist prescribed topical Soolantra (Ivermectin), which is specific for the inflammation characteristic of rosacea. At the same time, she took various over-the-counter treatments for ‘gut health’ as her IBS-C continued to cause her distress and her diet was restricted by intolerance to various foods, primarily carbohydrates. After 6 months, she discontinued the prescription ointment as her skin had not responded. In 2021, she experienced the onset of ocular rosacea in the right eye. This resulted in corneal opacity, leading to blurred vision and the onset of blood vessels in the sclera of that eye. The ophthalmologist prescribed oral antibiotics and steroid eye drops to reduce the severe burning and stinging as the patient described her symptoms. She persevered for 6 months to no avail.

**Figure 8:**
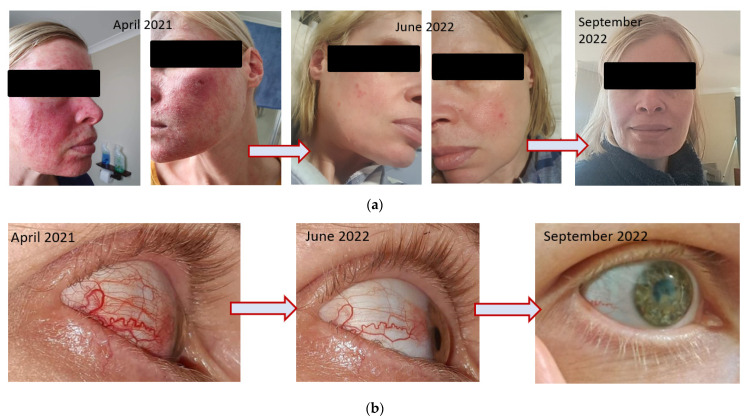
(**a**)A 4-year history of Facial Rosacea in a 40-year-old woman. Visual improvement of the condition from April 2021 until September 2022 is apparent in the photographs. (**b**) The same patient with photographic progress of Ocular Rosacea across 6 months.

She began treatment with SFN similar to that described in the earlier cases, and by September 2022, 6 months later, she had little to no facial or ocular rosacea (Figure 8a,b). She was able to increase the diversity of her diet, including the ability to tolerate oats, cashews and multiple types of fruit. Her Bristol Stool Score reflected the improvement in IBS-C constipation (Bristol 1–2 to 3–4). Figure 8a,b illustrate the progress of both the facial and the ocular lesions.

## 10. Conclusions

The detailed investigation of SFN has extended globally over a 30-year period, during which in vitro, animal and human interventional trials have established its ability to influence a range of biochemical processes, many of which significantly influence the expression of genes critical to upstream cellular processes. SFN is a phytochemical that is both potent as an Nrf2 activator and is sufficiently bioavailable that some in vitro findings readily translate to the clinical environment.

This author, as an experienced clinician in nutritional medicine with a research background in phytochemicals with significant clinical potential, has observed the effects of SFN in humans ingesting it by way of a myrosinase-active broccoli sprout supplement. What appeared initially to be an adverse reaction in some people ingesting SFN now appears to have been in part due to the ability of this molecule to influence gut ecology, possibly by initiating the release of endogenous antimicrobials from the specialised intestinal Paneth cells. Whereas many clinicians use pharmaceutical and non-pharmaceutical antimicrobials to eradicate a pathogen or pathobiont from the gut lumen, this author considers it possible that SFN may be capable of selectively achieving this end without simultaneously compromising the natural gut inhabitants, the commensal micro-organisms.

From this observation evolved the hypothesis put forward in this article, which subsequently led to the exploration of SFN’s clinical application in humans. As a functional food with a demonstrated safety profile, the composition of whole broccoli sprout materials would not per se be expected to exhibit any effects that might compromise core biochemical processes, even though it may produce unpleasant symptoms in some individuals. For clinicians experienced in recommending SFN to patients, initial adverse gut effects may be qualitatively considered to be semi-diagnostic of a microbial imbalance that can be addressed with appropriate dose titration.

With its foundational goal of restoring homeostasis to the gut ecosystem, this hypothesis has been incorporated into clinical interventions that utilise SFN as part of comprehensive dietary recommendations. For the last four years, the author has been teaching other clinicians in nutritional therapy how to implement this approach, which we describe as Gut Ecology and Metabolic Modulation. Although its initial focus was on the conditions affecting the digestive system, it soon became apparent that comorbid systemic issues were also responsive.

As with any hypothesis, there is much yet to be learned and to be tested; many unanswered questions remain, not the least of which are those described in Section 4. In seeking an effective gateway for addressing digestive, immune, cardiometabolic and other chronic disease, this hypothesis proposes an approach that harnesses the endogenous processes of human cells. These processes focus on restoring homeostasis to the gut, its underlying immune network, and the companion microbiota, with the collective potential to beneficially impact all gut–organ axes.

## Data Availability

Given that the original data for these observational case studies were provided by clinicians from their confidential records, no external data repository is available.

## References

[1] Meldrum J.M. (1993). What is nutritional medicine?. Nutr. Health.

[2] Sperber A.D., Bangdiwala S.I., Drossman D.A., Ghoshal U.C., Simren M., Tack J., Whitehead W.E., Dumitrascu D.L., Fang X., Fukudo S. (2021). Worldwide Prevalence and Burden of Functional Gastrointestinal Disorders, Results of Rome Foundation Global Study. Gastroenterology.

[3] Sperber A.D., Dumitrascu D., Fukudo S., Gerson C., Ghoshal U.C., Gwee K.A., Hungin A.P.S., Kang J.-Y., Minhu C., Schmulson M. (2017). The global prevalence of IBS in adults remains elusive due to the heterogeneity of studies: A Rome Foundation working team literature review. Gut.

[4] Soncini M., Stasi C., Satta P.U., Milazzo G., Bianco M., Leandro G., Montalbano L.M., Muscatiello N., Monica F., Galeazzi F. (2019). IBS clinical management in Italy: The AIGO survey. Dig. Liver Dis..

[5] Hawrelak J.A., Cattley T., Myers S.P. (2009). Essential oils in the treatment of intestinal dysbiosis: A preliminary in vitro study. Altern. Med. Rev..

[6] Valdes A.M., Walter J., Segal E., Spector T.D. (2018). Role of the gut microbiota in nutrition and health. BMJ.

[7] Shapiro T.A., Fahey J.W., Dinkova-Kostova A.T., Holtzclaw W.D., Stephenson K.K., Wade K.L., Ye L., Talalay P. (2006). Safety, tolerance, and metabolism of broccoli sprout glucosinolates and isothiocyanates: A clinical phase I study. Nutr. Cancer.

[8] Houghton C.A., Fassett R.G., Coombes J.S. (2013). Sulforaphane: Translational research from laboratory bench to clinic. Nutr. Rev..

[9] Ahlawat S., Asha, Sharma K. (2021). Gut–organ axis: A microbial outreach and networking. Lett. Appl. Microbiol..

[10] Turnbaugh P.J., Ley R.E., Hamady M., Fraser-Liggett C.M., Knight R., Gordon J.I. (2007). The Human Microbiome Project. Nature.

[11] Leech B., Schloss J., Steel A. (2019). Treatment Interventions for the Management of Intestinal Permeability: A Cross-Sectional Survey of Complementary and Integrative Medicine Practitioners. J. Altern. Complement Med..

[12] Khan R., Petersen F.C., Shekhar S. (2019). Commensal Bacteria: An Emerging Player in Defense Against Respiratory Pathogens. Front. Immunol..

[13] Maier L., Goemans C.V., Wirbel J., Kuhn M., Eberl C., Pruteanu M., Müller P., Garcia-Santamarina S., Cacace E., Zhang B. (2021). Unravelling the collateral damage of antibiotics on gut bacteria. Nature.

[14] Thapa D., Losa R., Zweifel B., Wallace R.J. (2012). Sensitivity of pathogenic and commensal bacteria from the human colon to essential oils. Microbiology.

[15] Sasso J.M., Ammar R.M., Tenchov R., Lemmel S., Kelber O., Grieswelle M., Zhou Q.A. (2023). Gut Microbiome-Brain Alliance: A Landscape View into Mental and Gastrointestinal Health and Disorders. ACS Chem. Neurosci..

[16] Shulpekova Y.O., Nechaev V.M., Popova I.R., Deeva T.A., Kopylov A.T., Malsagova K.A., Kaysheva A.L., Ivashkin V.T. (2021). Food Intolerance: The Role of Histamine. Nutrients.

[17] Panacer K., Whorwell P.J. (2019). Dietary Lectin exclusion: The next big food trend?. World J. Gastroenterol.

[18] Gray P.E., Mehr S., Katelaris C.H., Wainstein B.K., Star A., Campbell D., Joshi P., Wong M., Campbell D., Joshi P. (2013). Salicylate elimination diets in children: Is food restriction supported by the evidence?. Med. J. Aust..

[19] Bellini M., Tonarelli S., Nagy A.G., Pancetti A., Costa F., Ricchiuti A., de Bortoli N., Mosca M., Marchi S., Rossi A. (2020). Low FODMAP Diet: Evidence, Doubts, and Hopes. Nutrients.

[20] Petroski W., Minich D.M. (2020). Is There Such a Thing as "Anti-Nutrients"? A Narrative Review of Perceived Problematic Plant Compounds. Nutrients.

[21] Vojdani A., Afar D., Vojdani E. (2020). Reaction of Lectin-Specific Antibody with Human Tissue: Possible Contributions to Autoimmunity. J. Immunol. Res..

[22] Hrubisko M., Danis R., Huorka M., Wawruch M. (2021). Histamine Intolerance-The More We Know the Less We Know. A Review. Nutrients.

[23] Ermer T., Nazzal L., Tio M.C., Waikar S., Aronson P.S., Knauf F. (2023). Oxalate homeostasis. Nat. Rev. Nephrol..

[24] Ali A., Tan H., Kaiko G.E. (2020). Role of the Intestinal Epithelium and Its Interaction with the Microbiota in Food Allergy. Front. Immunol..

[25] Peterson L.W., Artis D. (2014). Intestinal epithelial cells: Regulators of barrier function and immune homeostasis. Nat. Rev. Immunol..

[26] Allaire J.M., Crowley S.M., Law H.T., Chang S.-Y., Ko H.-J., Vallance B.A. (2018). The Intestinal Epithelium: Central Coordinator of Mucosal Immunity. Trends Immunol..

[27] Houghton C.A., Fassett R.G., Coombes J.S. (2016). Sulforaphane and Other Nutrigenomic Nrf2 Activators: Can the Clinician’s Expectation Be Matched by the Reality?. Oxidative Med. Cell. Longev..

[28] Houghton C.A. (2019). Sulforaphane: Its “Coming of Age” as a Clinically Relevant Nutraceutical in the Prevention and Treatment of Chronic Disease. Oxidative Med. Cell. Longev..

[29] David L.A., Maurice C.F., Carmody R.N., Gootenberg D.B., Button J.E., Wolfe B.E., Ling A.V., Devlin A.S., Varma Y., Fischbach M.A. (2014). Diet rapidly and reproducibly alters the human gut microbiome. Nature.

[30] Sanders M.E., Merenstein D., Merrifield C.A., Hutkins R. (2018). Probiotics for human use. Nutr. Bull..

[31] Grazul H., Kanda L.L., Gondek D. (2016). Impact of probiotic supplements on microbiome diversity following antibiotic treatment of mice. Gut Microbes.

[32] Bezkorovainy A. (2001). Probiotics: Determinants of survival and growth in the gut. Am. J. Clin. Nutr..

[33] Zangara M.T., McDonald C. (2019). How diet and the microbiome shape health or contribute to disease: A mini-review of current models and clinical studies. Exp. Biol. Med..

[34] Suez J., Zmora N., Zilberman-Schapira G., Mor U., Dori-Bachash M., Bashiardes S., Zur M., Regev-Lehavi D., Brik R.B.-Z., Federici S. (2018). Post-Antibiotic Gut Mucosal Microbiome Reconstitution Is Impaired by Probiotics Improved by Autologous FMT. Cell.

[35] Walter J. (2008). Ecological role of lactobacilli in the gastrointestinal tract: Implications for fundamental and biomedical research. Appl. Environ. Microbiol..

[36] Canny G., Swidsinski A., McCormick B.A. (2006). Interactions of intestinal epithelial cells with bacteria and immune cells: Methods to characterize microflora and functional consequences. Methods Mol. Biol..

[37] Donaldson G.P., Lee S.M., Mazmanian S.K. (2016). Gut biogeography of the bacterial microbiota. Nat. Rev. Microbiol..

[38] Litvak Y., Byndloss M.X., Baumler A.J. (2018). Colonocyte metabolism shapes the gut microbiota. Science.

[39] Okumura R., Takeda K. (2017). Roles of intestinal epithelial cells in the maintenance of gut homeostasis. Exp. Mol. Med..

[40] Abreu M.T. (2010). Toll-like receptor signalling in the intestinal epithelium: How bacterial recognition shapes intestinal function. Nat. Rev. Immunol..

[41] Zhang X., Shu X.-O., Xiang Y.-B., Yang G., Li H., Gao J., Cai H., Gao Y.-T., Zheng W. (2011). Cruciferous vegetable consumption is associated with a reduced risk of total and cardiovascular disease mortality. Am. J. Clin. Nutr..

[42] Talalay P., Fahey J.W. (2001). Phytochemicals from cruciferous plants protect against cancer by modulating carcinogen metabolism. J. Nutr..

[43] Yagishita Y., Fahey J.W., Dinkova-Kostova A.T., Kensler T.W. (2019). Broccoli or Sulforaphane: Is It the Source or Dose That Matters?. Molecules.

[44] Yagishita Y., Gatbonton-Schwager T.N., McCallum M.L., Kensler T.W. (2020). Current Landscape of NRF2 Biomarkers in Clinical Trials. Antioxidants.

[45] Zhang Y., Talalay P., Cho C.G., Posner G.H. (1992). A major inducer of anticarcinogenic protective enzymes from broccoli: Isolation and elucidation of structure. Proc. Natl. Acad. Sci. USA.

[46] Fahey J.W., Zhang Y., Talalay P. (1997). Broccoli sprouts: An exceptionally rich source of inducers of enzymes that protect against chemical carcinogens. Proc. Natl. Acad. Sci. USA.

[47] Moi P., Chan K., Asunis I., Cao A., Kan Y.W. (1994). Isolation of NF-E2-related factor 2 (Nrf2), a NF-E2-like basic leucine zipper transcriptional activator that binds to the tandem NF-E2/AP1 repeat of the beta-globin locus control region. Proc. Natl. Acad. Sci. USA.

[48] Cuadrado A., Rojo A.I., Wells G., Hayes J.D., Cousin S.P., Rumsey W.L., Attucks O.C., Franklin S., Levonen A.-L., Kensler T.W. (2019). Therapeutic targeting of the NRF2 and KEAP1 partnership in chronic diseases. Nat. Rev. Drug Discov..

[49] He F., Ru X., Wen T. (2020). NRF2, a Transcription Factor for Stress Response and Beyond. Int. J. Mol. Sci..

[50] Cuadrado A., Manda G., Hassan A., Alcaraz M.J., Barbas C., Daiber A., Ghezzi P., León R., López M.G., Oliva B. (2018). Transcription Factor NRF2 as a Therapeutic Target for Chronic Diseases: A Systems Medicine Approach. Pharmacol. Rev..

[51] Bahadoran Z., Mirmiran P., Hosseinpanah F., Hedayati M., Hosseinpour-Niazi S., Azizi F. (2011). Broccoli sprouts reduce oxidative stress in type 2 diabetes: A randomized double-blind clinical trial. Eur. J. Clin. Nutr..

[52] Surh Y.J., Kundu J.K., Na H.K. (2008). Nrf2 as a master redox switch in turning on the cellular signaling involved in the induction of cytoprotective genes by some chemopreventive phytochemicals. Planta Med..

[53] Macdonald T.T., Monteleone G. (2005). Immunity, inflammation, and allergy in the gut. Science.

[54] Thimmulappa R.K., Mai K.H., Srisuma S., Kensler T.W., Yamamoto M., Biswal S. (2002). Identification of Nrf2-regulated genes induced by the chemopreventive agent sulforaphane by oligonucleotide microarray. Cancer Res..

[55] Wardyn J.D., Ponsford A.H., Sanderson C.M. (2015). Dissecting molecular cross-talk between Nrf2 and NF-κB response pathways. Biochem. Soc. Trans..

[56] Kirpich I.A., Parajuli D., McClain C.J. (2015). The gut microbiome in NAFLD and ALD. Clin. Liver Dis..

[57] Velloso L.A., Folli F., Saad M.J. (2015). TLR4 at the Crossroads of Nutrients, Gut Microbiota, and Metabolic Inflammation. Endocr. Rev..

[58] Eren E., Tufekci K.U., Isci K.B., Tastan B., Genc K., Genc S. (2018). Sulforaphane Inhibits Lipopolysaccharide-Induced Inflammation, Cytotoxicity, Oxidative Stress, and miR-155 Expression and Switches to Mox Phenotype through Activating Extracellular Signal-Regulated Kinase 1/2-Nuclear Factor Erythroid 2-Related Factor 2/Antioxidant Response Element Pathway in Murine Microglial Cells. Front. Immunol..

[59] Subedi L., Lee J.H., Yumnam S., Ji E., Kim S.Y. (2019). Anti-Inflammatory Effect of Sulforaphane on LPS-Activated Microglia Potentially through JNK/AP-1/NF-kappaB Inhibition and Nrf2/HO-1 Activation. Cells.

[60] Fahey J.W., Stephenson K.K., Wade K.L., Talalay P. (2013). Urease from Helicobacter pylori is inactivated by sulforaphane and other isothiocyanates. Biochem. Biophys. Res. Commun..

[61] Tobin I., Zhang G. (2023). Regulation of Host Defense Peptide Synthesis by Polyphenols. Antibiotics.

[62] Youn H.S., Kim Y.S., Park Z.Y., Kim S.Y., Choi N.Y., Joung S.M., Seo G.A., Lim K.-M., Kwak M.K., Hwang D.H. (2010). Sulforaphane suppresses oligomerization of TLR4 in a thiol-dependent manner. J. Immunol..

[63] Jeon M., Lee J., Lee H.K., Cho S., Lim J.-H., Choi Y., Pak S., Jeong H.-J. (2020). Sulforaphane mitigates mast cell-mediated allergic inflammatory reactions in in silico simulation and in vitro models. Immunopharmacol. Immunotoxicol..

[64] Jadkauskaite L., Bahri R., Farjo N., Farjo B., Jenkins G., Bhogal R., Haslam I., Bulfone-Paus S., Paus R. (2018). Nuclear factor (erythroid-derived 2)-like-2 pathway modulates substance P-induced human mast cell activation and degranulation in the hair follicle. J. Allergy Clin. Immunol..

[65] López-Chillón M.T., Carazo-Díaz C., Prieto-Merino D., Zafrilla P., Moreno D.A., Villaño D. (2019). Effects of long-term consumption of broccoli sprouts on inflammatory markers in overweight subjects. Clin. Nutr..

[66] Hanlon N., Coldham N., Gielbert A., Kuhnert N., Sauer M.J., King L.J., Ioannides C. (2007). Absolute bioavailability and dose-dependent pharmacokinetic behaviour of dietary doses of the chemopreventive isothiocyanate sulforaphane in rat. Br. J. Nutrition..

[67] Ristow M., Zarse K., Oberbach A., Klöting N., Birringer M., Kiehntopf M., Stumvoll M., Kahn C.R., Blüher M. (2009). Antioxidants prevent health-promoting effects of physical exercise in humans. Proc. Natl. Acad. Sci. USA.

[68] Barrett K.E., Minor J.R., Metcalfe D.D. (1985). Histamine secretion induced by N-acetyl cysteine. Agents Actions.

[69] Leitner R., Zoernpfenning E., Missbichler A. (2014). Evaluation of the inhibitory effect of various drugs/active ingredients on the activity of human diamine oxidase in vitro. Clin. Transl. Allergy.

[70] Hermsdorff H.H., Zulet M.A., Puchau B., Martinez J.A. (2010). Fruit and vegetable consumption and proinflammatory gene expression from peripheral blood mononuclear cells in young adults: A translational study. Nutr. Metab..

[71] Li W., Khor T.O., Xu C., Shen G., Jeong W.S., Yu S., Kong A.N. (2008). Activation of Nrf2-antioxidant signaling attenuates NFkappaB-inflammatory response and elicits apoptosis. Biochem. Pharmacol..

[72] Piotrowska M., Swierczynski M., Fichna J., Piechota-Polanczyk A. (2021). The Nrf2 in the pathophysiology of the intestine: Molecular mechanisms and therapeutic implications for inflammatory bowel diseases. Pharmacol. Res..

[73] Chelakkot C., Ghim J., Ryu S.H. (2018). Mechanisms regulating intestinal barrier integrity and its pathological implications. Exp. Mol. Med..

[74] Horowitz A., Chanez-Paredes S.D., Haest X., Turner J.R. (2023). Paracellular permeability and tight junction regulation in gut health and disease. Nat. Rev. Gastroenterol. Hepatol..

[75] Ulluwishewa D., Anderson R.C., McNabb W.C., Moughan P.J., Wells J.M., Roy N.C. (2011). Regulation of tight junction permeability by intestinal bacteria and dietary components. J. Nutr..

[76] Leech B., McIntyre E., Steel A., Sibbritt D. (2019). Risk factors associated with intestinal permeability in an adult population: A systematic review. Int. J. Clin. Pract..

[77] De Santis S., Cavalcanti E., Mastronardi M., Jirillo E., Chieppa M. (2015). Nutritional Keys for Intestinal Barrier Modulation. Front. Immunol..

[78] Davis W., Emmaus P.A. (2012). Lose the Wheat, Lose the Weight: Banish Your Wheat Belly, Feel Better Than Ever, and Turbocharge Your Health.

[79] Choung R.S., Unalp-Arida A., Ruhl C.E., Brantner T.L., Everhart J.E., Murray J.A. (2016). Less Hidden Celiac Disease But Increased Gluten Avoidance Without a Diagnosis in the United States: Findings From the National Health and Nutrition Examination Surveys From 2009 to 2014. Mayo Clinic Proceedings.

[80] Golley S., Corsini N., Topping D., Morell M., Mohr P. (2015). Motivations for avoiding wheat consumption in Australia: Results from a population survey. Public. Health Nutr..

[81] Thaiss C.A., Levy M., Grosheva I., Zheng D., Soffer E., Blacher E., Braverman S., Tengeler A.C., Barak O., Elazar M. (2018). Hyperglycemia drives intestinal barrier dysfunction and risk for enteric infection. Science.

[82] Houghton C.A., Ghosh D. (2021). Chapter 14-The gut microbiome: Its role in brain health. Nutraceuticals in Brain Health and Beyond.

[83] Cantorna M.T., Snyder L., Arora J. (2019). Vitamin A and vitamin D regulate the microbial complexity, barrier function, and the mucosal immune responses to ensure intestinal homeostasis. Crit. Rev. Biochem. Mol. Biol..

[84] Gleeson J.P. (2017). Diet, food components and the intestinal barrier. Nutr. Bull..

[85] El Khoury D., Balfour-Ducharme S., Joye I.J. (2018). A Review on the Gluten-Free Diet: Technological and Nutritional Challenges. Nutrients.

[86] Lerner A., Matthias T. (2015). Changes in intestinal tight junction permeability associated with industrial food additives explain the rising incidence of autoimmune disease. Autoimmun. Rev..

[87] Moonwiriyakit A., Pathomthongtaweechai N., Steinhagen P.R., Chantawichitwong P., Satianrapapong W., Pongkorpsakol P. (2023). Tight junctions: From molecules to gastrointestinal diseases. Tissue Barriers.

[88] Lechuga S., Ivanov A.I. (2017). Disruption of the epithelial barrier during intestinal inflammation: Quest for new molecules and mechanisms. Biochim. Biophys. Acta Mol. Cell Res..

[89] Fischbach M.A., Segre J.A. (2016). Signaling in Host-Associated Microbial Communities. Cell.

[90] Sanlier N., Gokcen B.B., Sezgin A.C. (2019). Health benefits of fermented foods. Crit. Rev. Food Sci. Nutr..

[91] Beck B.R., Park G.S., Lee Y.H., Im S., Jeong D.Y., Kang J. (2019). Whole Genome Analysis of Lactobacillus plantarum Strains Isolated from Kimchi and Determination of Probiotic Properties to Treat Mucosal Infections by Candida albicans and Gardnerella vaginalis. Front. Microbiol..

[92] Pascal M., Perez-Gordo M., Caballero T., Escribese M.M., Longo M.N.L., Luengo O., Manso L., Matheu V., Seoane E., Zamorano M. (2018). Microbiome and Allergic Diseases. Front. Immunol..

[93] Rooks M.G., Garrett W.S. (2016). Gut microbiota, metabolites and host immunity. Nat. Rev. Immunol..

[94] Zheng D., Liwinski T., Elinav E. (2020). Interaction between microbiota and immunity in health and disease. Cell Res..

[95] Malard F., Dore J., Gaugler B., Mohty M. (2021). Introduction to host microbiome symbiosis in health and disease. Mucosal Immunol..

[96] Burgueño J.F., Abreu M.T. (2020). Epithelial Toll-like receptors and their role in gut homeostasis and disease. Nat. Rev. Gastroenterol. Hepatol..

[97] Hug H., Mohajeri M.H., La Fata G. (2018). Toll-Like Receptors: Regulators of the Immune Response in the Human Gut. Nutrients.

[98] Rezac S., Kok C.R., Heermann M., Hutkins R. (2018). Fermented Foods as a Dietary Source of Live Organisms. Front. Microbiol..

[99] McFarland L.V., Karakan T., Karatas A. (2021). Strain-specific and outcome-specific efficacy of probiotics for the treatment of irritable bowel syndrome: A systematic review and meta-analysis. EClinicalMed..

[100] Boyle R.J., Robins-Browne R.M., Tang M.L. (2006). Probiotic use in clinical practice: What are the risks?. Am. J. Clin. Nutr..

[101] Khalesi S., Bellissimo N., Vandelanotte C., Williams S., Stanley D., Irwin C. (2019). A review of probiotic supplementation in healthy adults: Helpful or hype?. Eur. J. Clin. Nutr..

[102] Hirose Y., Murosaki S., Yamamoto Y., Yoshikai Y., Tsuru T. (2006). Daily intake of heat-killed Lactobacillus plantarum L-137 augments acquired immunity in healthy adults. J. Nutr..

[103] Hirose Y., Yamamoto Y., Yoshikai Y., Murosaki S. (2013). Oral intake of heat-killed Lactobacillus plantarum L-137 decreases the incidence of upper respiratory tract infection in healthy subjects with high levels of psychological stress. J. Nutr. Sci..

[104] Pique N., Berlanga M., Minana-Galbis D. (2019). Health Benefits of Heat-Killed (Tyndallized) Probiotics: An Overview. Int. J. Mol. Sci..

[105] Johnston D.G., Corr S.C. (2016). Toll-Like Receptor Signalling and the Control of Intestinal Barrier Function. Methods Mol. Biol..

[106] Mukhopadhyay S., Sekhar K.R., Hale A.B., Channon K.M., Farrugia G., Freeman M.L., Gangula P.R. (2011). Loss of NRF2 impairs gastric nitrergic stimulation and function. Free Radic. Biol. Med..

[107] Brandl K., Kumar V., Eckmann L. (2017). Gut-liver axis at the frontier of host-microbial interactions. Am. J. Physiol. Gastrointest. Liver Physiol..

[108] Macfarlane S., Bahrami B., Macfarlane G.T. (2011). Mucosal biofilm communities in the human intestinal tract. Adv. Appl. Microbiol..

[109] Fahey J.W., Haristoy X., Dolan P.M., Kensler T.W., Scholtus I., Stephenson K.K., Talalay P., Lozniewski A. (2002). Sulforaphane inhibits extracellular, intracellular, and antibiotic-resistant strains of Helicobacter pylori and prevents benzo[a]pyrene-induced stomach tumors. Proc. Natl. Acad. Sci. USA.

[110] Haristoy X., Fahey J.W., Scholtus I., Lozniewski A. (2005). Evaluation of the antimicrobial effects of several isothiocyanates on Helicobacter pylori. Planta Med..

[111] Yanaka A., Fahey J.W., Fukumoto A., Nakayama M., Inoue S., Zhang S., Tauchi M., Suzuki H., Hyodo I., Yamamoto M. (2009). Dietary sulforaphane-rich broccoli sprouts reduce colonization and attenuate gastritis in Helicobacter pylori-infected mice and humans. Cancer Prev. Res..

[112] Ghoshal U., Shukla R., Srivastava D., Ghoshal U.C. (2016). Irritable Bowel Syndrome, Particularly the Constipation-Predominant Form, Involves an Increase in Methanobrevibacter smithii, Which Is Associated with Higher Methane Production. Gut Liver.

[113] Inoue R., Sakaue Y., Kawada Y., Tamaki R., Yasukawa Z., Ozeki M., Ueba S., Sawai C., Nonomura K., Tsukahara T. (2019). Dietary supplementation with partially hydrolyzed guar gum helps improve constipation and gut dysbiosis symptoms and behavioral irritability in children with autism spectrum disorder. J. Clin. Biochem. Nutr..

[114] Helmick C.G., Lee-Han H., Hirsch S.C., Baird T.L., Bartlett C.L. (2014). Prevalence of psoriasis among adults in the U.S.: 2003–2006 and 2009-2010 National Health and Nutrition Examination Surveys. Am. J. Prev. Med..

[115] Oliveira Mde F., Rocha Bde O., Duarte G.V. (2015). Psoriasis: Classical and emerging comorbidities. Bras. Dermatol..

